# Tetraspanins regulate cell-to-cell transmission of HIV-1

**DOI:** 10.1186/1742-4690-6-64

**Published:** 2009-07-14

**Authors:** Dimitry N Krementsov, Jia Weng, Marie Lambelé, Nathan H Roy, Markus Thali

**Affiliations:** 1 Department of Microbiology and Molecular Genetics, University of Vermont, Burlington, VT 05405, USA; 2 Graduate Program in Microbiology and Molecular Genetics, University of Vermont, Burlington, VT 05405, USA; 3 Graduate Program in Cellular and Molecular Biology, University of Vermont, Burlington, VT 05405, USA

## Abstract

**Background:**

The presence of the tetraspanins CD9, CD63, CD81 and CD82 at HIV-1 budding sites, at the virological synapse (VS), and their enrichment in HIV-1 virions has been well-documented, but it remained unclear if these proteins play a role in the late phase of the viral replication cycle. Here we used overexpression and knockdown approaches to address this question.

**Results:**

Neither ablation of CD9, CD63 and/or CD81, nor overexpression of these tetraspanins was found to affect the efficiency of virus release. However, confirming recently reported data, tetraspanin overexpression in virus-producing cells resulted in the release of virions with substantially reduced infectivity. We also investigated the roles of these tetraspanins in cell-to-cell transmission of HIV-1. Overexpression of CD9 and CD63 led to reduced cell-to-cell transmission of this virus. Interestingly, in knockdown experiments we found that ablation of CD63, CD9 and/or CD81 had no effect on cell-free infectivity. However, knockdown of CD81, but not CD9 and CD63, enhanced productive particle transmission to target cells, suggesting additional roles for tetraspanins in the transmission process. Finally, tetraspanins were found to be downregulated in HIV-1-infected T lymphocytes, suggesting that HIV-1 modulates the levels of these proteins in order to maximize the efficiency of its transmission within the host.

**Conclusion:**

Altogether, these results establish an active role of tetraspanins in HIV-1 producer cells.

## Background

Persistence of HIV-1 in infected individuals is a major public health problem. Despite great advances in anti-retroviral therapies, the virus cannot be completely eliminated once infection is established. One (of the many) potential explanation(s) for this failure of infected individuals to clear the virus is that its mode of spread does not allow components of the immune system to recognize and attack it appropriately. It is now well documented that HIV-1 can be transferred very efficiently from cell-to-cell, most likely upon induction of so-called virological synapses (VSs), sites of transient adhesion between infected (producer) and uninfected (target) cells [1-7]. Upon formation of the VS, viral budding is polarized towards the target cell, and the virus is thought to be released into the tight synaptic cleft where it appears to be at least partially protected from neutralizing antibodies [3].

Clearly, how the VS is formed and organized is an important question. Like immunological synapse (IS) formation, VS formation likely requires the concerted action of numerous cellular factors, several of which are also utilized for IS formation. Indeed it has been proposed that HIV-1 infection, followed by the expression of viral proteins, favors the formation of VSs at the expense of IS formation (e.g. [8], reviewed in [9]). However, we are only at a very early stage of understanding the relative distribution and function of viral and cellular elements during the formation, maintenance and disengagement of the VS. Specifically, while some studies show that VS formation and virus transfer is primarily driven by Env-CD4 interactions [2,3,10], other cellular components involved are only beginning to be unveiled.

Tetraspanins are a 33-member family of 4-span transmembrane proteins. They are thought to act as membrane organizers, selectively clustering proteins into microdomains in order for specific membrane-based processes to proceed [11,12]. These processes include (among others) cell-cell fusion, cell adhesion, and cell motility (reviewed e.g. in [13,14]). Interestingly, tetraspanins are recruited to sites of HIV-1 budding, as evidenced by their incorporation into viral particles [15,16], as well as their clustering at budding sites in virus-producing cells, including the VS [17-22]. While several reports document involvement of these proteins in HIV-1 entry and possibly activation of newly infected cells [23-27], it remained unclear whether these proteins also play a functional role during the late stages of virus replication, specifically during the budding process [28-30]. We have recently shown that treatment of virus-producing HeLa cells with an anti-CD9 antibody reduces virus release [31]. While this pointed towards a potential role of this tetraspanin in HIV-1 budding, it appears now more likely that this treatment, which results in the clustering not only of CD9 but also of other tetraspanins and of all the viral structural components, simply redirects particle formation towards cell-cell contact sites, thus reducing the overall area through which progeny virus can exit from cells [32].

Here, we show that tetraspanins in producer cells, rather than acting as budding co-factors, regulate cell-free virus infectivity (confirming recent data by the Koyanagi laboratory [33]) and cell-to-cell transmission. Further, we provide evidence that HIV-1 modulates the levels of tetraspanins in producer cells in order to maximize the efficiency of its dissemination. Altogether, our findings identify new cellular players involved in the late stages of viral replication, specifically in the transfer of viral particles from cell-to-cell, a process that is crucial for HIV-1 dissemination in infected individuals.

## Methods

### Cell culture, plasmids, and antibodies

The following reagents were obtained through the NIH AIDS Research and Reference Reagent Program, Division of AIDS, NIAID, NIH: TZM-bl cells from Dr. John C. Kappes, Dr. Xiaoyun Wu and Tranzyme Inc, Jurkat Clone E6-1 cells from Dr. Arthur Weiss, CEM.NKR-CCR5-Luc (referred to as CEM-Luc throughout) cells from Drs. John Moore and Catherine Spenlehauer, CEM-GFP cells from Dr. Jacques Corbeil; as well as reverse transcriptase inhibitors Zidovudine and Efavirenz, and the following antibodies: monoclonal antibody to HIV-1 p24 (AG3.0) from Dr. Jonathan Allan, HIVIG from NABI and NHLBI, HIV-1 p24 Hybridoma (183-H12-5C) from Dr. Bruce Chesebro.

HeLa and TZM-bl cells were maintained in DMEM with 10% FBS. All T cell lines were maintained in RPMI with 10% FBS. Media for CEM-GFP and CEM.NKR-CCR5-Luc were supplemented with 0.5 and 0.8 mg/ml G418, respectively.

The following proviral plasmids were used: pNL4-3, pNL4-3deltaNef (provided by Dr. John Guatelli, UCSD), HIV-i-GFP (pNL4-3 containing a MA-GFP fusion, and an additional protease cleavage site between MA and GFP) [34], and HXB2 (provided by Dr. Clarisse Berlioz-Torrent, Institut Cochin, France). Previously described [33], tetraspanin and L6 expression plasmids (in pCMV-Sport6 vector) were provided by Dr. Koyanagi, (Kyoto University, Japan). The FG12 shRNA delivery vector system was developed in Dr. David Baltimore's laboratory [35] and was obtained via the AddGene service (addgene.org).

### Tetraspanin knockdown

ShRNA sequences were based on previously described siRNA sequences: [24] for CD9 and CD81, and Ambion's pre-designed CD63 (ID:10412). The scrambled shRNA sequence was generated by randomly scrambling the siRNA sequence used for CD63 silencing. Using NCBI BLAST, the scrambled sequence showed no significant homology with the human genome.

The lentiviral shRNA system used has been previously described [35], and cloning and other procedures were carried out essentially as described. Briefly, stock lentiviruses were produced by transfecting 293T cells with the FG12shRNA plasmids, pVSVG and packaging plasmid pDeltaR8.2. Supernatants were concentrated 200 fold by centrifugation at 50,000 g for 2 hours, then stored at -80°C. Titer was determined by infecting either HeLa or Jurkat cells with serial dilutions of virus, then determining %GFP positive cells by flow cytometry. Infections were done in the presence of 8 μg/ml polybrene (HeLa) or 10 μg/ml DEAE-dextran (Jurkat) to enhance transduction efficiency. Knockdown efficiency was confirmed by Western blot and flow cytometry, using the following antibodies: mouse anti-CD63, clone H5C6 (Developmental Studies Hybridoma Bank at the University of Iowa), mouse anti-CD9, clone K41 (Bachem), mouse anti-CD81, clone JS-81 (BD Biosciences), and mouse anti-GAPDH (Abcam). GAPDH was used as a loading control. Lysates were analyzed for protein content using the Coomassie Plus reagent (Pierce), and equal amounts of protein (5–10 μg) were loaded for each lane.

### HIV-1 release assays

HeLa cells were seeded at a density of 0.3 × 10^6 ^cells per well in 6 well plates (day 0). On day 1, cells were transduced with lentiviruses carrying shRNA at MOI = 8 overnight in the presence of 8 μg/ml Polybrene (Sigma). On day 3, cells were transfected with pNL4-3, using Lipofectamine2000 following manufacturer's instructions (Invitrogen). On day 5, supernatant containing released virus was centrifuged at 3,000 g, 10 minutes, to pre-clear, followed by 2 hours at 16,000 g through a 20% sucrose cushion. Viral pellets were re-suspended in TNE lysis buffer (10 mM Tris pH 7.4, 150 mM NaCl, 1 mM EDTA, 1% Triton-X100). Cells were lysed, centrifuged at 16,000 g for 10 minutes and were loaded for SDS-PAGE and Western blot together with released virus. AG3.0 anti-p24 antibody was used to detect Gag. Cell lysates and supernatants were also analyzed for p24 content by p24 antigen capture ELISA as described [36], with the following modifications: HIV-IG was used as an antigen detection antibody, followed by donkey anti-human HRP-conjugated antibody (Jackson ImmunoResearch) and a TMB substrate kit was used for colorimetric detection (Pierce).

### Cell-free infectivity assay

Supernatants from virus-expressing HeLa cells were pre-cleared at 3,200 g for 10 minutes, then assayed for p24 content using a home-made ELISA kit (see above). TZM-bl reporter cells, which express beta-galactosidase and luciferase under the control of the HIV LTR, were plated in 96-well plates (10,000 cells/well) one day prior to infection. 0.2 – 0.05 ng of p24 in 100 μl of media containing 20 μg/ml DEAE-dextran (to enhance infection) was used per well. 48 hours later, media was removed, cells were washed and 50 μl/well of All-in-One mammalian beta-galactosidase reagent (Pierce, USA) was added. The plate was incubated at 37°C for 5–30 minutes, and absorbance was read at 405 nm in a microplate reader. The absorbance was normalized by the p24 input for different dilutions and further normalized to the mock condition for that experiment (set to 1).

### Cell-to-cell transmission assay

#### Co-transfections

50,000 HeLa cells/well were plated in 24 well plates on Day 0. On Day 1, cells were co-transfected with tetraspanin expression plasmids and the appropriate proviral plasmid at 1:2 ratio. Transfections were performed in quadruplicate. On Day 2, media was removed, and target cells (either 0.2 million (mio) CEM-GFP or 0.4 mio CEM-Luc cells) were added to 2 out of the 4 quadruplicate wells. On Day 3, non-adherent target cells were removed and transferred to a new plate. Supernatants from replicate wells without target cells were harvested and assayed for p24 content using a home-made ELISA kit (used for normalization, see below). These supernatants were also used in infectivity assays (see above). On Day 4, non-adherent target cells were removed from the second plate. CEM-GFP cells were fixed with 4% paraformaldehyde (PFA) and analyzed by flow cytometry, the readout being %GFP positive cells. CEM-Luc cells were lysed in TNE Lysis buffer, then assayed for luciferase activity using the Bright-Glo Luciferase Assay System (Promega, USA). Both readouts for were normalized by p24 content of the replicate wells without target cells, and normalized to the mock condition.

#### Knockdowns

Cell-to-cell transmission assays with tetraspanin knockdowns were performed essentially as described above, with the following differences. HeLa cells were infected with lentiviruses carrying shRNA at an MOI = 8 2 days prior to transfection with pNL4-3, and the rest of the assay was carried out using CEM-Luc target cells as described above.

When Jurkat cells were used as producers, these were simultaneously infected with NL4-3 at an approximate MOI of 0.2 and with lentiviruses carrying shRNA at MOI = 8. 3 days post-infection, these cells were counted, 0.25 mio cells were mixed with 0.25 mio CEM-Luc target cells, and co-cultured for 3 days, or cultured without target cells. The cells were then lysed and assayed for luciferase activity as above. This readout was normalized by p24 output of producer cells without target cells to correct for potential cell death of producer cells due to shRNA treatment.

#### Latrunculin B treatment

The cell-to-cell transmission assay was carried out as above, with the following modifications. Producer HeLa cells were pretreated with Latrunculin B for 1 hour prior to addition of CEM-GFP target cells. Producer and target cells were co-cultured in the presence of Latrunculin B for 24 hours, target cells were removed and washed, then cultured for another 24 hours and analyzed by flow cytometry.

### Immunofluorescence microscopy

Jurkat cells were infected with stock NL4-3 virus at an approximate MOI = 0.1. 48 hours later, cells were allowed to adhere to Cell-Tak (BD Biosciences) coated coverslips in Mattek dishes (Mattek Corporation, USA) for 1–2 hours, then fixed and stained with tetraspanin and Gag antibodies. The following antibodies were used: mouse anti-CD63, clone H5C6 (Developmental Studies Hybridoma Bank at the University of Iowa), mouse anti-CD81, clone JS-81 (BD Biosciences), anti-p6 Gag rabbit serum (gift of Dr. David Ott, NCI Frederick). The cells were then incubated with appropriate AlexaFluor488 and AlexaFluor594 secondary antibodies (Invitrogen, USA). The slides were examined on a Delta Vision Workstation (DV base 3/3.5, Nikon Eclipse TE200 epifluorescence microscope fitted with an automated stage, Applied Precision Inc., Issaquah, WA, USA) and images were captured in z-series with a CCD digital camera (CoolSnap HQ). Out of focus light was digitally reassigned using Softworx deconvolution software (Applied Precision Inc.). Relative tetraspanin signal intensity was quantified using Volocity software (Improvision), using the classifier module set to 1 SD below the mean intensity (and no upper limit) to define the regions of interest, then analyzing their mean intensity. The background fluorescence intensity was also determined by selecting an area of a micrograph devoid of cells, and this value was subtracted from the mean intensity value determined for cells. At least 20 cells were analyzed for each condition.

#### Visualization of VS formation

Microscopy experiments were performed essentially as above, with the following modifications. Uninfected target Jurkat cells were labeled with CMAC CellTracker Blue (Invitrogen) according to manufacturer's instructions, then mixed with an equal number of infected producer cells. The cell mixture was incubated on Cell-Tak-coated coverslips for 2 hours at 37°C, then fixed, stained and visualized as above.

### Statistical analyses

A two-tailed, unequal variance Student's t-test was used to determine significance throughout. A p value less than 0.05 was considered significant.

## Results

### CD9, CD63 and CD81 are dispensable for HIV-1 budding

**Figure 1:**
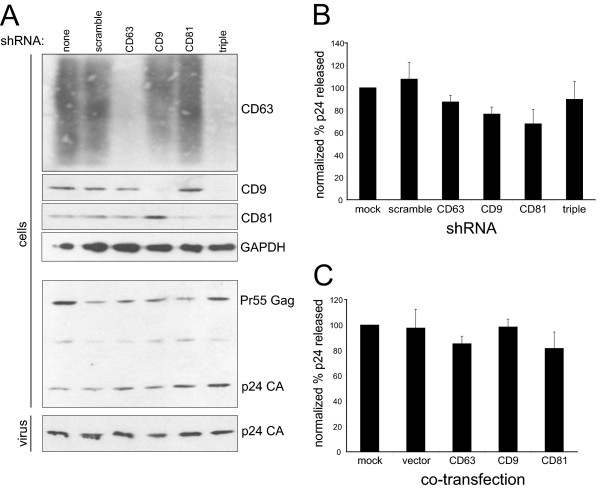
**Tetraspanins are not release factors**. HeLa cells were transduced with the indicated shRNA lentiviral vectors at MOI = 8, then transfected with pNL4-3 provirus 48 hours post-transduction. 2 days later, cells and viruses were harvested as described in Methods, and analyzed by Western blot for CD63, CD9, CD81 and Gag levels (A), or p24 ELISA for p24 CA content (B). Normalized percent release was calculated by dividing the amount of p24 released by the total amount of p24 produced (cells + supernatant), then normalizing to the mock-treated sample (set to 100%). Data shown represent the mean of 4 independent experiments, +/- standard error of the mean (SEM). (C) HeLa cells were co-transfected with pNL4-3 and the indicated plasmids. Cells and viruses were harvested and analyzed by p24 ELISA, as in (B). Data shown represent the mean of 5 independent experiments, +/- SEM.

Because tetraspanins are known to enhance or repress the function of peripheral and integral membrane proteins and because of their well-documented presence at HIV-1 exit sites, we tested if they act as budding co-factors. In order to achieve efficient silencing of tetraspanins, a lentivirus-based shRNA delivery system was employed. Using this system, at high MOI approximately 99% of the cells were transduced, and levels of CD63, CD9 and CD81 could be reduced dramatically (see Fig. 1A). Note that CD63 shows up as a smear on the Western blot, due to heavy glycosylation of this protein. Production of viral proteins and particle release in tetraspanin knock-down cells was compared to protein production and virus shedding in control cells, using Western blot and p24 ELISA (Figures 1A and 1B). As documented in Fig. 1, all three tetraspanins tested were dispensable for HIV-1 assembly and budding, as ablation of any one or all three of them did not significantly affect the efficiency of release (p > 0.05). Consistent with a recent report [33], overexpression of these three tetraspanins, which augmented their surface presence 5, 8, and 7 fold for CD9, CD63, and CD81, respectively, (see [37] for flow cytometry analysis), also did not affect particle release (Fig. 1C), although we noticed that CD81 overexpression decreased the synthesis of viral proteins (data not shown). Because of this variability in viral output, transmission and infectivity assay data were always corrected by the amount of virus released (see Methods).

### Overexpression of tetraspanins in HIV-1 producer cells reduces the infectivity of cell-free virus as well as cell-to-cell transmission of virions to target cells

**Figure 2:**
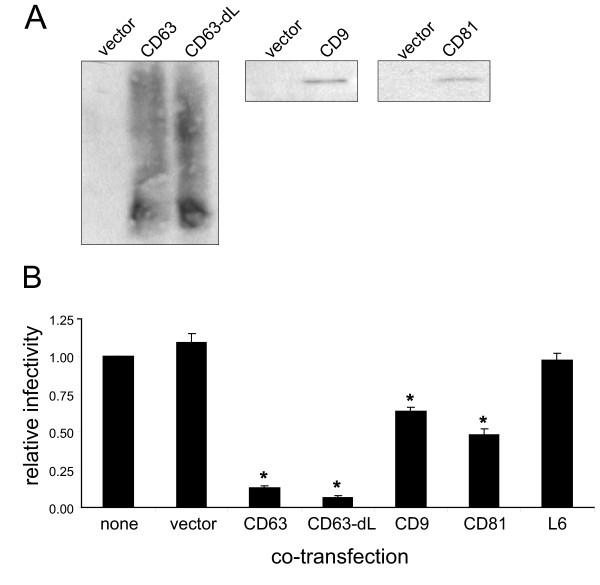
**Overexpression of tetraspanins decreases the infectivity of cell-free virus**. (A)HeLa cells were co-transfected with pNL4-3 and the indicated plasmids. Released virus was pelleted through a 20% sucrose cushion, immunoprecipitated using HIVIG and protein A sepharose beads (to purify the virus from tetraspanin-bearing exosomes or microvesicles), then eluted and analyzed for p24 content by ELISA. Equal amounts of p24 were loaded for each sample and analyzed for tetraspanin incorporation by Western blot. (B) HeLa cells were co-transfected with pNL4-3 and the indicated tetraspanin expression plasmids as in (A). Supernatants were collected, normalized for p24 content, and the infectivity assay was carried out as described in Methods. Data shown represent the mean of 5 independent experiments (normalized to vector control) +/- SEM. * denotes a statistically significant difference from vector control (p < 0.001).

Tetraspanins clearly are enriched in HIV-1 particles (for a recent review, [16]); yet as shown above, their presence at the viral exit site is not required for particle formation and release. We thus set out to test if the virus benefits from the presence of these membrane proteins once they are incorporated into virions. Contrary to our expectation, however, our initial data (not shown) clearly demonstrated that this is not the case, and indeed while work for this report was in progress, a study by Koyanagi and colleagues showed that overexpression of CD9, CD63, CD81 and other tetraspanins in virus-producing cells resulted in production of virions with considerably reduced infectivity [33]. As documented in Fig. 2, similar results were obtained in our system, which we adapted to use the same set of expression vectors used by the Koyanagi laboratory, in order to be able to directly compare the two studies. Thus, HeLa cells were co-transfected with the proviral expression plasmid pNL4-3 and tetraspanin expression plasmids. Cell-free supernatants from these producer cells that contained viral particles (equal amounts of p24 for each condition, normalized by p24 ELISA) were then used to infect TZM-bl cells, which contain the beta-galactosidase reporter gene controlled by the HIV LTR [38]. Co-transfection of HeLa cells with the proviral construct and with tetraspanin expressors, particularly with a CD63 expressor or with a plasmid that expressed a CD63 which lacks part of its internalization signal/lysosomal sorting motif (CD63delL), led to increased levels of tetraspanins incorporated into released virions (Fig. 2A), and this markedly reduced the infectivity of the virus (Fig. 2B). In contrast, co-transfection of a plasmid that expressed L6, a tetraspanin-related protein that is situated within the same plasma membrane microdomains [39], had no effect on the infectivity of the viral particles.

Reduced infectivity of cell-free virus may not necessarily translate into similarly reduced cell-to-cell transmission, since even particles with reduced affinity for their receptors, for example, may still successfully enter cells if they are shed into the cleft of the VS (i.e. directly next to the target cell). We thus sought to determine if the tetraspanin overexpression also negatively affects the infectivity of the virus in a cell-to-cell transmission system.

**Figure 3:**
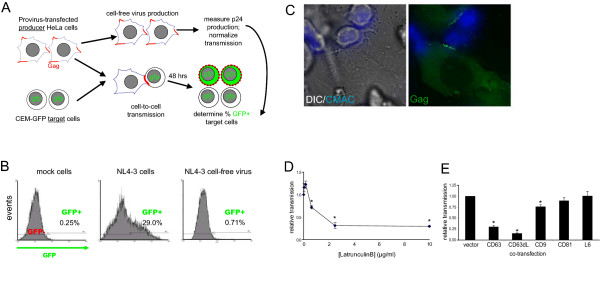
**Overexpression of CD63 and CD9 inhibits cell-to-cell transmission of HIV-1**. (A) A scheme for the cell-to-cell transmission assay. (B) Representative flow cytometry data from the cell-to-cell transmission assay. HeLa cells were transfected or not with pNL4-3, then co-cultured with CEM-GFP target cells. Alternatively, cell-free supernatant was collected from producer cells and used to infect CEM-GFP cells (free virus). (C) HeLa cells were transfected with HIV-i-GFP (pNL4-3 with MA-GFP, green), co-cultured with CMAC-labeled CEM cells (blue), and visualized by fluorescence microscopy. (D) pNL4-3-transfected HeLa cells were co-cultured with CEM-GFP targets cells in the presence of the indicated amount of Latrunculin B for 24 hrs. Target cells were removed, washed, cultured for another 24 hrs, and then analyzed by flow cytometry. (E) HeLa cells were co-transfected with pNL4-3 and the indicated tetraspanins. The cell-to-cell transmission assay was carried out as described in Methods. Data shown represent the mean of 7 independent experiments (normalized to vector control) +/- SEM. * denotes a statistically significant difference from vector control (p < 0.01).

Fig. 3A outlines the assay that was set up to monitor productive cell-to-cell transmission of HIV-1. In this system, HIV-1-producing HeLa cells are co-cultured with indicator CEM-GFP T lymphocytes, which express GFP under the control of the HIV LTR [40]. After 24 hours of co-culture, the target CEM-GFP cells were gently resuspended and removed, leaving behind adherent producer cells as well as producer-target syncytia, which were also adherent. Further, larger cells (potentially syncytia or detached HeLa cells) were excluded from the read-out of the assay based on side and forward scatter characteristics during the subsequent flow cytometry analysis. After another 24 hours of culture, the cells were again resuspended and removed from the culture flask, again leaving behind any adherent cells; they were then fixed and analyzed for GFP expression by flow cytometry. Importantly, we knew that most of the infection of the reporter cells resulted from their direct contact with producer cells, because hardly any productive infection could be detected if the target cells were incubated with equivalent amounts of cell-free virus (Fig. 3B). Further, HIV-1-producing HeLa cells formed VS-like contacts with CEM target cells, and cell-to-cell transmission could be blocked using Latrunculin B to depolymerize actin, as previously described in the T cell-T cell scenario [2,3] (see Figures 3C and 3D). Finally, this cell-to-cell transfer could be almost completely blocked by adding reverse transcriptase inhibitors (RTIs) AZT and Efavirenz (EFV) simultaneously with the addition of target cells (data not shown), demonstrating that reporter GFP expression was not a result of cell-cell fusion, which would be insensitive to RTIs, but rather would be the result of viral integration and productive infection.

As shown in Fig. 3E, using this cell-to-cell transmission assay, we found that over-expression of CD63 and to a lesser extent CD9, but not CD81, in producer cells, reduced productive virus transfer to target cells. The magnitude of the transmission reduction by CD63 overexpression was roughly half of the effect on cell-free virus infectivity. As measured for the infectivity of cell-free virus, overexpression of L6 had no effect on cell-to-cell virus transfer.

**Figure 4:**
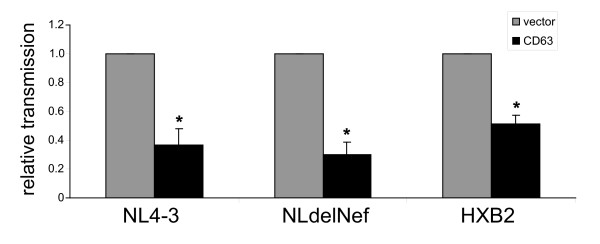
**Nef and Vpu are not required for inhibition of cell-to-cell transmission by CD63**. HeLa cells were co-transfected with the indicated proviral plasmid and either with pCD63 or empty vector. Cell-to-cell transmission assay was carried out as described in Methods. Data shown represent the mean of 3 independent experiments (normalized to vector control for each virus) +/- SEM. * denotes a statistically significant difference from vector control (p < 0.01).

Since HIV-1 accessory proteins Nef and Vpu are well-documented modulators of viral infectivity and release, respectively, we also tested if their presence was required for the suppression of cell-to-cell transfer by CD63 overexpression. HeLa cells were co-transfected with a CD63 expressor plasmid and either with pNL4-3deltaNef (NLdelNef) which gives rise to the production of virus that lacks Nef, or with an HXB2 proviral plasmid, which leads to the expression of HIV-1 lacking both Nef and Vpu. Fig. 4 documents that CD63 overexpression suppressed cell-to-cell transmission of both viruses, indicating that Nef and Vpu are dispensable for the reduced cell-to-cell transmission caused by this tetraspanin.

### Knockdown of CD81 enhances cell-to-cell transmission of HIV-1, but not the infectivity of cell-free virus

**Figure 5:**
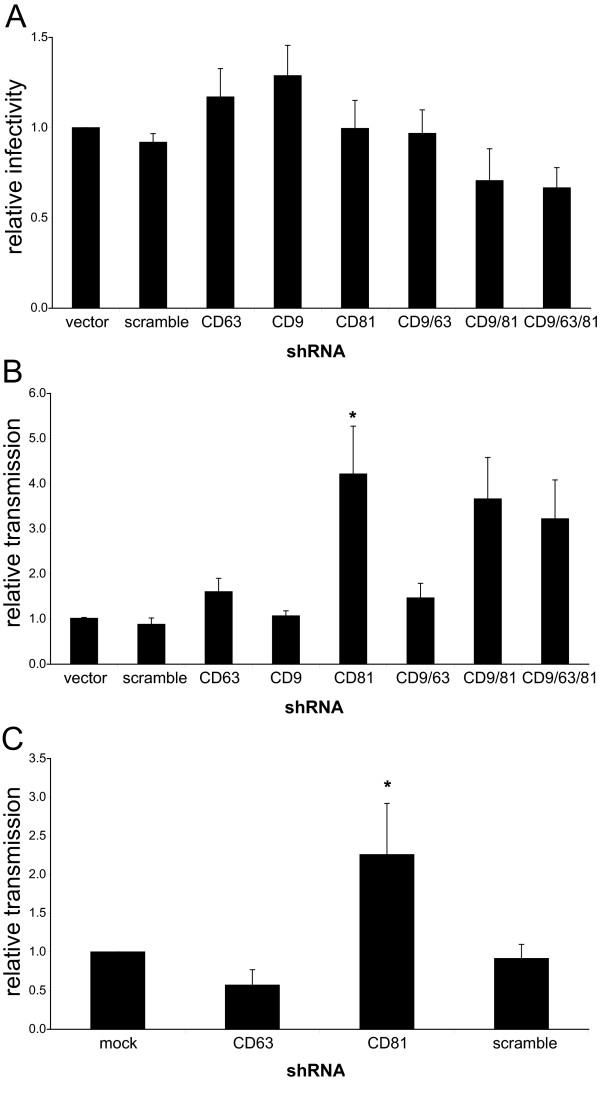
**Knockdown of CD81 enhances cell-to-cell transmission, but not cell-free infectivity**. HeLa cells were infected with the indicated shRNA-carrying lentiviruses and transfected with pNL4-3. (A) Cell-free infectivity. Data shown represent the mean of 7 independent experiments (normalized to vector control) +/- SEM. (B) Cell-to-cell transmission. Data shown represent the mean of 4 independent experiments (normalized to vector control) +/- SEM. (C) Jurkat T cells were co-infected with shRNA-carrying lentiviruses and NL4-3. Data shown represent the mean of 4 independent experiments (normalized to vector control) +/- SEM. Cell-to-cell transmission was carried out as described in Methods. * denotes a statistically significant difference from scrambled shRNA (p < 0.05).

The results shown in Figures 2 and 3 suggest that tetraspanins may play a restrictive role in HIV-1 replication. We thus hypothesized that knockdown of these host factors would enhance virion infectivity and cell-to-cell transmission. However, ablation of CD9, CD63 or CD81 in HIV-1-producing HeLa cells, either alone or pair wise, or even simultaneous knockdown of all three tetraspanins, had no effect on cell-free virus infectivity (Fig. 5A).

To measure cell-to-cell transmission initiated by tetraspanin-depleted producer cells, we had to modify the assay shown in Fig. 3 because the lentiviral system for shRNA delivery uses GFP as an indicator of transduction. Instead of CEM-GFP cells, we used CEM.NKR-CCR5-Luc (CEM-Luc) cells, which express luciferase under the control of the LTR [41], as targets. Surprisingly, only the knockdown of CD81, but not the ablation of the other tetraspanins, enhanced cell-to-cell transmission of HIV-1 (Fig. 5B). However, since this effect was not paralleled by a significant increase in the entry of cell-free virus into target cells (p > 0.05), as shown in Fig. 5A, we concluded that CD81 knockdown in producer cells must affect another step in cell-to-cell transmission of HIV-1.

Importantly, the lentivirus-based shRNA knockdown system allowed us to also assess the effects of tetraspanin ablation in T cells. Jurkat T lymphocytes were co-infected with high doses of NL4-3 virus and lentiviruses carrying shRNA against CD63 and CD81. CD9 knockdown was not performed, since E6-1 Jurkat cells express very low amounts of this protein, similar to primary CD4+ T cells [21]. These producer cells were then co-cultured with CEM-Luc target cells to allow for cell-to-cell transmission of virus. As in the HeLa system, knockdown of CD81 was found to enhance the cell-to-cell transmission of HIV-1 (Fig. 5C), without increasing cell-free virus infectivity (data not shown). Because this version of the transmission assay does not allow one to specifically exclude syncytia events, these experiments were also performed in the presence of EFV, to allow for syncytia formation but not successful transmission. As in previous experiments, EFV abolished most of the reporter activity, and the residual reporter activity did not change significantly in either of the knockdown conditions (data not shown).

### Tetraspanins are downregulated in acutely infected lymphocytes

**Figure 6:**
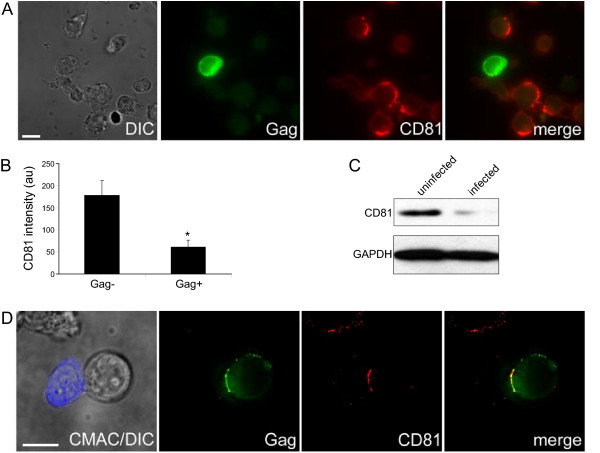
**CD81 is downregulated in acutely infected lymphocytes**. Jurkat T lymphocytes were infected with NL4-3, then fixed and stained for Gag and CD81. (A) A representative image of CD81 labeling in infected Jurkat cells. A single Z-section is shown. Scale bar represents 10 μm. (B) Quantification of immuno-fluorescence labeling of CD81 in infected (Gag+) and uninfected (Gag-) cells. Data shown represent the mean +/- SEM. * denotes a statistically significant difference from uninfected cells (p < 0.05). See Methods for more details. (C) Jurkat cells were infected with NL4-3. 4 days post-infection, when the percentage of infected cells was above 50%, cells were lysed, and CD81 and GAPDH protein levels were analyzed by Western blot. (D) Recruitment of CD81 to the VS in acutely infected Jurkat cells. Target Jurkat cells were labeled with CMAC Cell-tracker (blue) and co-cultured with NL4-3-infected producer Jurkat cells for 2 hours on PLL-coated coverslips. The cells were then fixed and stained for the indicated antigens as described in Methods. A single Z-section is shown. Maximum and minimum intensity of each wavelength were adjusted in order to highlight the areas with the most intense signal. Scale bars represent 10 μm.

HIV-1 has been well documented to downregulate various cellular factors that are detrimental to its replication, such as CD4 or MHC Class I (reviewed e.g. in [42]). Since CD63, CD9, and CD81 appear to play partially restrictive roles in HIV-1 replication by reducing the infectivity of cell-free virions and also by inhibiting cell-to-cell transmission, it seemed plausible that the virus would adapt to overcome this restriction. Indeed, in cultures of infected Jurkat lymphocytes lower levels of CD81 could be readily observed by fluorescence microscopy analysis of newly infected cells expressing high amounts of Gag (Fig. 6A and 6B). A similar downregulation could be observed by quantitative Western blot in HIV-1-infected Jurkat cells (Fig. 6C). We also measured similar downregulation of CD82, another tetraspanin that is abundantly expressed in T lymphocytes (data not shown), and of CD9 and CD63, although these were more difficult to analyze, particularly in Jurkat sub-clones that have low endogenous levels of these two tetraspanins (data not shown). However, no tetraspanin downregulation was detected in provirus-transfected HeLa cells (data not shown). Finally, although the levels of tetraspanins were reduced in HIV-1-infected T cells, their recruitment to and their concentration at the VS were still readily detectable (Fig. 6D for CD81, data not shown for CD9 and CD63), suggesting that these proteins are not eliminated completely, and may still carry out certain functions (e.g. cell-cell fusion prevention, [37]).

## Discussion

While tetraspanins have been well-documented to be involved in various steps of the HIV-1 replication cycle, until recently only evidence for their functional involvement in potential target cells and in newly infected cells has been presented. Here, we provide data which establish tetraspanins also as regulators of HIV-1 spread through their presence in HIV-1-producing cells.

Despite the enrichment of CD63, CD9 and CD81 at HIV-1 budding sites, their knockdown did not affect the efficiency of HIV-1 release from HeLa cells. This suggests that these proteins do not act as budding factors for HIV-1, although we cannot formally exclude the possibility of compensation by other functionally redundant tetraspanins (e.g. CD82, CD151, etc). These results are in line with findings of the Marsh group, who reported that knockdown of CD63 in macrophages did not alter the efficiency of viral release, regardless of whether the knockdown was done prior to or before infection [30], though another recent study reported that CD63 knockdown after HIV-1 infection of macrophages reduced the amount of virus released from these cells [28]. However, in the latter study, it remained unclear exactly which step was inhibited by CD63 ablation, as the amounts of released particles apparently were not corrected by the levels of cell-associated viral proteins. Further, another recent study utilizing a chronically infected T-cell line reported that knockdown of CD81 or treatment with an anti-CD81 antibody reduced viral release [29]. It is possible that this effect may be dependent on the cell type, and the potential release functions of tetraspanins may not be recapitulated in the HeLa cell system.

Overexpression of CD63, CD9 and CD81 repressed cell-free virus infectivity. Similar data were recently published by Koyanagi and colleagues [33], although in our study the magnitude of the repression appears to vary more between different tetraspanins, the most potent being CD63. However, while it seems likely that specific tetraspanins act differently, we cannot exclude that this effect simply depends on endogenous levels of the tetraspanin at the surface in producer cells, with CD63 displaying the lowest surface fraction of the tetraspanins analyzed in the cells used in our analysis. Koyanagi and colleagues also showed that the inhibition of infectivity was not due to altered Gag or Env processing or Env incorporation, and occurred specifically at the virus-cell fusion stage of entry. Consistent with the latter finding, we observed that cell-cell fusion driven by Env is also inhibited by tetraspanin overexpression [37].

As shown in Fig. 3, overexpression of CD63, and to a lesser extent of CD9, also inhibited cell-to-cell transmission of HIV-1, suggesting that reduced infectivity of virus is capable of reducing its cell-to-cell transmission. The magnitude of this repression was about half of the effect on cell-free infectivity, thus the effects on cell-free infectivity are not exactly matched in cell-to-cell transmission. It is quite possible that tetraspanin overexpression, in addition to suppressing infectivity, could also have other, potentially positive effects on transmission, e.g. preventing fusion of target and producer cells, and thus preserving productive VSs [37]. We have measured syncytia formation directly in our cell-to-cell transmission assay and found that while overexpression of CD63 repressed syncytia formation and transmission with similar efficiency, syncytia formation was relatively rare, occurring approximately once per 10 successful transmission events (data not shown), explaining why no obvious positive effect of syncytia repression was observed in our assays. Interestingly, overexpression of a different tetraspanin, CD82, was also shown to inhibit cell-to-cell transmission of another human retrovirus, HTLV-I, while also inhibiting syncytia formation [43]. CD82 and CD81 were found to associate with HTLV-I Env and/or Gag protein, suggesting that tetraspanins are part of a common pathway of fusion and transmission regulation for different retroviruses [43-45].

The fact that knockdown of CD63, CD9 and CD81 failed to increase cell-free infectivity, although in agreement with previously published data [30,33], is surprising, given that overexpression of these proteins reduced infectivity considerably. The most parsimonious explanation for this finding may be that the endogenous levels of these proteins in producer HeLa cells or acutely infected T cells are too low to effectively suppress infectivity. In fact, we have observed that uninfected lymphocytes have a relatively higher surface density of some tetraspanins, such as CD81 and CD82 (data not shown), and this may explain why the virus would downregulate these proteins to a level that is optimal for its infectivity, and yet is not too low to prevent syncytia formation (discussed in more detail below). Consistent with this, we have observed that virus-producing HeLa cells do not exhibit any appreciable tetraspanin downregulation (data not shown). Also, downregulation of tetraspanins in producer HeLa cells does in fact enhance syncytia formation [37]. Finally, Muriaux and colleagues reported that CD81 knockdown in chronically infected T cells reduced infectivity of released virus [29]. As above, we speculate that the levels of CD81 (and other tetraspanins) in these cells are higher than in HeLa cells or acutely infected or activated T cells, and this is supported by the findings of Koyanagi and colleagues [33].

Also consistent with the idea that the levels of CD9 and CD63 in HeLa cells are relatively low, downregulation of these two tetraspanins does not alter cell-to-cell transmission of HIV-1. Intriguingly though, knockdown of CD81 enhanced cell-to-cell virus transmission without affecting infectivity. This suggests that CD81 may play a role at a different step in the transmission process. Since this tetraspanin has been shown to be involved in cell-cell adhesion and cell migration, in addition to cell-cell fusion, it is possible that it affects the frequency or the duration of VS formation. Future studies will help to dissect exactly which step CD81 is involved in.

We found that tetraspanins are downregulated in infected lymphocytes, suggesting that HIV-1 may be actively lowering the levels of these proteins. This is in disagreement with data published by Sattentau and colleagues, who did not find a difference in CD63, CD9 and CD81 levels between infected and non-infected lymphocytes [21]. However, in the aforementioned study, viral proteins were not directly measured together with tetraspanin levels. In our experiments, we observed that tetraspanin expression was quite variable in infected cultures, and clear-cut downregulation was only observed in cells expressing relatively high levels of Gag. Interestingly, a similar downregulation of tetraspanins was observed in PHA/PMA-stimulated lymphocytes chronically infected with HIV-1, which also correlated with increased virus production and enhanced virion infectivity [33]. Intriguingly, enhancement of virion infectivity by HIV-1 Nef in CD4-negative cells has been recently shown to be dependent on the clathrin/dynamin2-dependent pathway [46]. It is tempting to speculate that HIV-1 Nef may be involved in downregulating tetraspanins from the plasma membrane, preventing their incorporation into virions and thus enhancing virion infectivity. Another possibility is that tetraspanins are simply shed in the released virions, as a consequence of their incorporation into virus particles, although experiments performed in our laboratory indicate that tetraspanin downregulation does not correlate with the amount of released virus (M.L., D.K. and M.T., unpublished data).

## Conclusion

Overall then, are tetraspanins restriction factors for HIV-1? Given that tetraspanins appear to reduce virus infectivity and cell-to-cell transmission, and that the virus downregulates these proteins, it is tempting to speculate that they indeed hinder HIV-1 replication. However, since tetraspanins also play a positive role, by preventing syncytia formation [37], and thus likely preserving the VS, it seems likely that the most beneficial scenario for the virus is to achieve a balance between enhanced infectivity and low syncytia formation. Consistent with this, we observed only a partial downregulation of tetraspanins in infected cells, and tetraspanins were still recruited to the VS (although at presumably lower levels, hence allowing for the enhancement of infectivity), where they could prevent cell-cell fusion. Further, while our current cell-to-cell transmission assays indicate a restrictive role for tetraspanins, this may not be the case *in vivo*, where fusion prevention may potentially be more important than infectivity enhancement. As discussed above, in our transmission assay, syncytia formation is a relatively rare event, occurring in roughly once per 10 successful transmission events (using HeLa cells as producers); thus a reduction in syncytia formation (e.g. by tetraspanin overexpression) would result in a very modest transmission enhancement. *In vivo *however, e.g. when the virus replicates in lymph nodes, where the cells are much more densely packed, there is less opportunity for producer and target cells to separate after VS formation and virus transmission, and the effects of impaired fusion regulation, if quantifiable, might become evident. Finally, another limitation of our transmission assays is that they measure the effect of tetraspanins on a single transmission step. However, multi-step transmission assays (e.g. a spreading infection in a lymphocyte culture), become very difficult to interpret, because tetraspanins, as mentioned before, also play a role in target cells.

Altogether, our results provide evidence for an active role of tetraspanins in cell-to-cell transmission of HIV-1, a process that may be crucial for virus spread *in vivo *and one which is only beginning to be understood. Because individual tetraspanins are not essential for host cell survival, they may provide an avenue for therapeutic intervention with the virus life cycle.

## Abbreviations

HIV-1: human immunodeficiency virus type 1; VS: virological synapse; GFP: green fluorescent protein; Env: HIV-1 envelope glycoprotein; MHC: major histocompatibility complex; SEM: standard error of the mean; PFA: paraformaldehyde; shRNA: small hairpin RNA.

## Competing interests

The authors declare that they have no competing interests.

## Authors' contributions

DNK, JW, and MT designed experiments and analyzed data. DNK performed most of the experiments. ML performed the experiments shown in Figure 6C. NHR. designed and cloned the shRNA constructs, and optimized the shRNA-mediated knockdowns. DNK and MT wrote the manuscript.
